# Exploring the Prevalence of Functional Gastrointestinal Diseases and the Accompanied Differences in Dietary and Lifestyle Patterns: A Two-Generational Study

**DOI:** 10.3390/diagnostics14151630

**Published:** 2024-07-29

**Authors:** Elham A. Aljaaly, Mai A. Khatib

**Affiliations:** 1 Department of Clinical Nutrition, Faculty of Applied Medical Sciences, King Abdulaziz University, P.O. Box 80200, Jeddah 21589, Saudi Arabia; ealjaaly@kau.edu.sa; 2 Medical Nutrition Therapy Unit, King Abdulaziz University Hospital, King Abdulaziz University, P.O. Box 80215, Jeddah 21589, Saudi Arabia; 3 Food, Nutrition, and Lifestyle Unit, King Fahd Medical Research Centre, King Abdulaziz University, P.O. Box 80216, Jeddah 21589, Saudi Arabia; 4 Obesity and Lifestyle Unit, King Abdulaziz University Hospital, King Abdulaziz University, P.O. Box 80215, Jeddah 21589, Saudi Arabia

**Keywords:** functional gastrointestinal diseases, COVID-19, generations, complementary and alternative medicines

## Abstract

Background and Objectives: Generation Z and millennials in Saudi Arabia both experienced the stress of the COVID-19 pandemic and the accompanying factors that may have had an impact on the incidence of functional gastrointestinal diseases (FGIDs) in both generations. This study aims to explore how prevalent FGIDs are among adolescents and their parents. Methods and Study Design: A cross-sectional, school-based study conducted in public high schools for boys and girls in Jeddah, Saudi Arabia. We adapted 37 items from the ROME IV Diagnostic Questionnaires for children and adults, as well as other questionnaires. IBM SPSS Statistics (Version 28.0) was used. Results: Generation Z showed a higher prevalence of FGIDs (33.5%, *n* = 126) in comparison with millennials (20.0%, *n* = 28). In both generations, the most prevalent FGID was functional constipation; the least prevalent were irritable bowel syndrome and abdominal migraine, with no significant change in the severity or frequency of symptoms during the pandemic. The type of commonly consumed beverages was a risk factor for FGIDs. Participants in generation Z were less likely to use complementary and alternative medicine (67.4%) to prevent diseases and enhance immunity compared with millennials (82.9%). Conclusions: The study results confirmed disparities in the prevalence of FGIDs between the two generations before and during the COVID-19 pandemic, which requires further research in other areas of Saudi Arabia. Recognizing the differences between the millennial parents and the generation Z high schoolers could assist health professionals in planning individualized, generation-based interventions and educators in designing and tailoring programs based on generational differences.

## 1. Introduction

Functional gastrointestinal disorders (FGIDs), or disorders of gut–brain interaction, are diagnosed noninvasively using the Rome Diagnostic Questionnaire (DQ), which inquires about symptoms and severity [1,2,3]. Saudi studies have shown that FGIDs are common in both females and males. Half of the women in one study had a minimum of one FGID [4]. Functional constipation was reported to have the highest prevalent among children in Saudi Arabia, compared with other countries [5]. The prevalence of functional constipation and functional abdominal pain was found to be 8.1% and 6.2%, respectively, among 319 children aged 3 to 18 years in the country’s Central Region [6]. However, the two conditions were less prevalent in a similar population in the Western Region [7], and a higher prevalence rate was reported for functional constipation among preschoolers (5%, 3 out of 59 children) [8]. According to Saudi reports, risk factors for FGIDs included age, sex, dietary factors, marital status, living situation, parental educational level, smoking, sleeping pattern, consumption of carbonated drinks, and infection with COVID-19 [4,6,9]. Clinical studies have shown that COVID-19 patients reported having gastrointestinal symptoms [10], and the prevalence of FGIDs was found to be higher in children during the pandemic when compared with studies conducted prior to the pandemic [11].

Considering the impact of generation on health, generational studies provide insights into key findings from recent studies exploring the relationship between generational characteristics and the prevalence of FGIDs. Recent reports reveal that generation Z has an increased awareness of and concern for environmental issues [12,13]. This generational feature becomes remarkably significant when considering studies investigating the connection between FGID symptoms and partner attitudes. Some studies have even noticed differences in the prevalence of FGID symptoms across different generations [14]. Living in a world of movement, the current two Saudi generations of parents and high schoolers grew up experiencing different environmental and life events. Their lifestyles, habits, and perspectives are different, but they both experienced the stressful times and anxiety, fear, sadness, and loneliness that resulted from the lockdown enforced during the COVID-19 pandemic. All those factors may have had an impact on the incidence of FGIDs in both generations. This study investigates the prevalence of FGIDs and the accompanying differences in dietary and lifestyle patterns in the current two Saudi generations: millennials (parents born between 1981 and 1996) and generation Z (high schoolers born between 1997 and 2012). The study also explores how this influence may be affected by the ongoing impact of COVID-19.

## 2. Materials and Methods

### 2.1. Study Design

This study is part of a larger study exploring differences in dietary habits and lifestyle between the two current generations before and during the COVID-19 pandemic. This cross-sectional, school-based study using an electronic self-reported survey was conducted in public high schools for boys and girls in Jeddah, Saudi Arabia, from September 2021 to April 2022.

### 2.2. Participants and Sampling

The sample included male and female students aged 15 to 18 years (grades 10 through 12) enrolled during the academic year 2021–2022 in public education high schools whose parents resided in Jeddah city. We excluded students outside the specified age range and students with all medically diagnosed gastrointestinal diseases.

**Figure 1:**
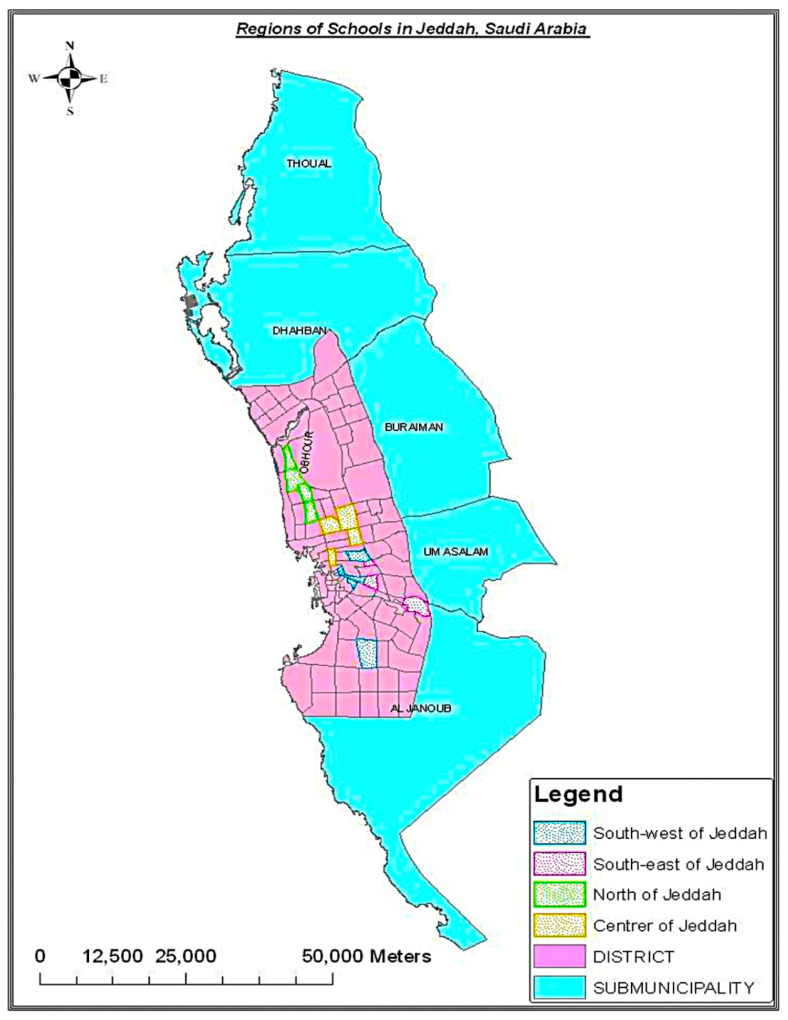
Map of school districts in Jeddah city. Source: Aljaaly (2012) [16].

**Figure 2:**
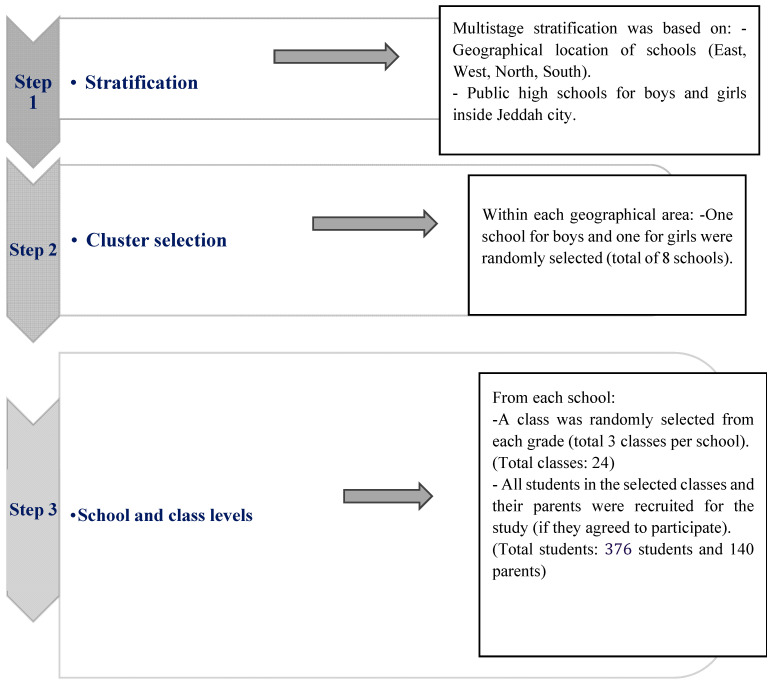
Sample selection criteria and participant recruitment.

The Epi Info calculator was used to identify the required sample size, considering the total number of high school students in Jeddah to be 153,641 students [15]. Based on an estimated dropout rate of 20%, a 95% confidence level, a 5% error margin, and a design effect of 1, the estimated sample size was 245 students. After doubling that number to include parents, the total sample size needed to be 490 (245 students and 245 parents). Based on previously published works on sample selection in similar populations (Figure 1) [16] and the Ministry of Education’s school districts for Jeddah city (Eastern, Western, Northern, Southern) at the time of data collection (Table 1) [17], participants were selected through their schools by multistage random sampling. The first stage was to select an equal number of students from each school district. The second stage was to randomly select two schools (one for boys and one for girls) from each district. Three classes (one from each grade) from each school were randomly selected and enrolled in the study (average 41–60 students per class). Each class was considered a cluster, and all the students in that class and their parents were invited to participate in the study (Figure 2).

### 2.3. Data Collection and Study Tool

An online self-administered questionnaire was developed, with permission, guided by the literature on epidemiological studies and surveillance of FGIDs, generational studies of students’ and their parents’ food patterns, lifestyle, and infection with COVID-19 [18,19,20], and Rome foundation criteria [21]. The study tool was adapted from the Rome IV DQ for adolescents and adults and included other questions to define participant demographics and assess food and lifestyle patterns. An expert panel of eight health professionals with medical and clinical nutrition backgrounds reviewed, edited, and approved the questionnaire. The questionnaire included four sections. The first section comprised 11 questions on sociodemographic characteristics. The second section, adapted from both Rome IV DQs, was concerned with the diagnosis and prevalence of FGIDs. The third and fourth sections evaluated food and lifestyle patterns as well as changes during the pandemic, including dietary patterns, sleep patterns, and physical activity.

After fine-tuning, the questionnaire was shared online and tested in a pilot study on a sample of 94 individuals (58 students and 36 parents) using the same recruitment criteria. The aim of the pilot study, conducted two months prior to this one, was to examine and refine the measurement instrument.

### 2.4. Statistical Analysis

Frequency and percentage are used in this report to present sociodemographic variables. Scoring of questions on FGIDs followed Rome Foundation guidelines. Identifying risk factors and significant differences in food and lifestyle patterns between the two generations and before and after the pandemic was carried out using chi-square test. Associations between different factors and exploration of predictors for FGIDs were carried out using multivariate regression analysis. There were no missing data, because the Google Forms toolrequires mandatory responses to all questions before continuing through the survey. Significance level was set at a *p* value of 0.05 or below. IBM SPSS Statistics (Version 28) was used to analyze the data.

### 2.5. Ethical Approval and Consent to Participate

The Research Ethics Committee of the Faculty of Applied Medical Sciences at King Abdulaziz University granted the study approval on 2 November 2021, under the reference number FAMS-EC2021-15. Conducting the study in schools was approved by the Ministry of Education’s directorate of schools in Jeddah and principals of the selected schools. All participants signed an electronic consent form before proceeding to answer survey questions. To confirm clear and thorough reporting of the approach and findings of this work, the authors followed the STROBE checklist [22].

## 3. Results

Eight schools (4 each for girls and boys) with a total of 24 classes were included. The questionnaires were completed by 516 participants (140 parents (27.1%) and 376 students (72.9%)). The parent group consisted of 47 men (33.6%) and 93 women (66.4%), and the student group comprised 147 males (39.1%) and 229 females (60.9%). Participants in the parent group were aged 30 years and over (41 parents, 29.2%, were between 30 and 40 years old and 99 parents, 70.7%, were aged 41 or older), while the students were aged between 15 and 19 years. Thirty-two parents (22.9%) and 36 students (9.6%) reported having chronic diseases, and 41 parents (29.3%) and 100 students (26.6%) reported infection with COVID-19.

### 3.1. Prevalence of Functional Gastrointestinal Disorders

Twenty-eight parents (20.0%) and 126 students (33.5%) were classified as having FGIDs. In both generations, functional constipation was the most prevalent FGID, followed by functional abdominal pain. The least prevalent FGIDs were abdominal migraine and irritable bowel syndrome (Table 2). Moreover, both groups showed no significant change in severity or frequency of symptoms during the pandemic (*p* > 0.05).

### 3.2. Relationship Between FGIDs and Lifestyle Habits and COVID-19

The parent group showed no significant associations between FGID prevalence and the seven variables related to lifestyle and dietary behavior (*p* > 0.05) (Table 3 and Table 4). Conversely, significant associations were found between the prevalent FGIDs and commonly consumed meals (homemade vs. ready), number of takeout meals, and type of commonly consumed beverage, *p* < 0.05 in all cases of the students group (Table 5 and Table 6). However, the significance only extended to the type of commonly consumed beverage (sugary) in the multinomial regression analysis (*p* = 0.01; odds ratio (OR) = 1.18, confidence interval (CI) = 1.03–1.35) (Table 7).

Moreover, no significant association was found between the prevalence of FGIDs and infection with COVID-19 in either the parent or student group (*p* = 0.57, *p* = 0.26, respectively) (Table 3 and Table 5). Participants in both groups reported having FGIDs before the pandemic.

### 3.3. Practice of Complementary and Alternative Medicine

The results showed that 116 parents (82.9%) practiced more CAM as compared with 252 students (67.4%), *p* < 0.01. The most common reason reported for practicing CAM in both groups was to prevent diseases and enhance immunity. Moreover, 71 parents (50.7%) practiced more CAM in response to COVID-19 as compared with 109 students (29.0%), *p* < 0.01.

## 4. Discussion

The main objective of this study was to highlight the prevalence of FGIDs and the accompanying differences in food and lifestyle patterns among millennials and generation Z. A key novel finding is that functional constipation was the most common FGID among the two generations and that type of consumed beverages affected the prevalence of FGIDs most in generation Z. Twenty percent of millennials and 33.5% of individuals in generation Z were classified as having FGIDs. These findings are in line with reports in the literature, which showed that the prevalence of FGIDs in Saudi Arabia ranged between 9% and 40% [4,6,7,23,24]. The variation of prevalence rates from different parts of the country suggests a multifactorial etiology.

In the present study, the prevalence of FGIDs was strongly associated with some food and lifestyle patterns, such as commonly consumed meals (homemade vs. ready meals), number of takeout meals per day, and type of commonly consumed beverages. Takeout food, ready meals, and sugary beverages are generally energy dense, high in fats, added salts, and sugars, and low in fiber, vitamins, and minerals [25,26,27], all of which contribute to disease mortality and morbidity [26,27]. In the context of FGIDs, there have been limited reports of a link between the prevalence of FGIDs and negative dietary habits. In Kundur et al. study, individuals who regularly consumed fatty meals and fast food had a higher prevalence of FGIDs compared with the healthy group [4]. This finding is in line with similar observations regarding fried foods [28,29,30]. The link can be related to the fact that consumption of a lot of fatty and fast foods triggers the development of reflux symptoms by reducing esophageal sphincter pressure, increasing exposure of the esophagus to gastric juices and, thus, increasing gut sensitivity and irritation and causing functional upper gastrointestinal disorders [30,31,32]. Moreover, previous research has highlighted the influence of high energy meals on gastrointestinal transit time, causing disturbances and deregulation of proper digestion and leading to constipation [33,34,35,36]. These associations were found to be significant in generation Z but not millennials. A similar trend was seen previously, with higher prevalence of FGIDs in the younger group when compared with the older group [4], which is to be expected logically, as such negative lifestyle habits are known to be common among younger generations [37].

On the other hand, the incidence of FGIDs among millennials, although lower when compared with generation Z, suggests a different association that was not intended to be studied currently. Kundur et al. found a link between age and the incidence of FGIDs, which can be explained physiologically [4]. For instance, acid reflux and gas formation increase with age [4,38,39]. Addressing these associations in different age groups is important, as it can direct both the health care provider and patients on how to manage symptoms and inform the role of dietetics on what nutritional advice should be followed [40,41,42,43].

Of note in the present study is the finding that functional constipation was among the most prevalent FGID in both generations and that commonly consuming sugary drinks was the only lifestyle habit that contributed significantly to the prevalence of FGIDs. It is well documented in the literature that functional constipation is highly prevalent among people of different age groups in Saudi Arabia, due to a variety of discussed contributing causes [5,6,7,8,44,45,46]. Moreover, a positive association between many gastrointestinal problems, such as constipation, and sugary dietary items, specifically refined sugars, has been established previously [33,34,35,36,47,48]. Sugary drinks are mostly rich in refined sugars and energy and low in fiber [47]. There have been hypotheses discussing the complex relationship between functional constipation and the gut microbiota, which, in this case, can be affected by lifestyle habits, stressors, and diet [49]. Dysbiosis, which often refers to disruption in the gut microbiota, has been linked to increased rates of FGIDs including functional constipation [50,51,52]. Indeed, data from animal models have supported the interplay between regulation of gastrointestinal motility and the gut microbiome through complex metabolic and neuroendocrine mechanisms that are driven by dietary metabolites, such as short-chain fatty acids [53,54,55]. These hypotheses support the link between low fiber intake and, consequently, high sugar intake, and functional constipation, which explains the results of the present study and others [4]. However, whether there is a cause-and-effect relationship is still not fully understood given the inherent risk of bias related to the observational study design.

Observations have shown that the incidence of FGIDs was higher among COVID-19 patients when compared with healthy controls [56,57]. Some of the reports suggested “post-infectious” mechanisms, including direct viral invasion of the gastrointestinal tract, increased fecal calprotectin, presence of viral RNA in feces, altered intestinal permeability, gut microbiota dysbiosis, mucosal damage on gastrointestinal endoscopy, and involvement of enteric nervous system mechanisms that lead to FGID development [10]. However, the present findings showed no difference in FGID prevalence before and after the COVID-19 pandemic or before and after participants were infected with COVID-19. In fact, the participants reported having FGIDs before being infected with COVID-19, which explains the discrepancy with the literature. More research is needed to clarify the nature of this relationship.

Out of interest, the authors were keen to examine differences between the two generations in how important they regarded CAM, which is commonly practiced among people in Saudi Arabia and other countries [58,59,60,61,62]. In the present study, 82.9% of millennials and 67.4% of participants in generation Z practiced CAM to prevent diseases and enhance immunity. Moreover, 50.7% of millennials and 29.0% of those in generation Z reported practicing CAM in response to COVID-19. However, no significant relationship was found between practicing CAM and FGID symptom alleviation. This result comes contrary to theories in the literature, as recent review articles concluded that significant benefits were shown in patients with FGIDs following the use of some herbal therapies [63,64]. Future studies focusing on testing this link are required.

The key novelty in this study is exploring the generational difference in prevalence of FGIDs and related risk factors including food habits, lifestyle, and COVID-19 in both men and women. The use of the validated ROME IV DQ has made identification of FGIDs in larger samples much easier and more reliable. Nevertheless, data collection was carried out by inviting the students directly and their parents indirectly. Hence, participation was greater from the students than the parents. Nevertheless, limitations of this study include reliance on self-reporting at a single point in time to assess dietary and lifestyle behaviors and the use of an online and anonymous questionnaire. Moreover, it would be interesting for future research to consider the use of public toilets while at school, since the current results showed that functional constipation was the most common FGID. Furthermore, future research is should also be directed to comparing the current results to the COVID-19 and non-COVID-19 periods and to evaluate the depression symptoms that are commonly associated with anxiety during stressful period of times, such as the COVID-19 pandemic and its impact on FGIDs.

## 5. Conclusions

In conclusion, this study showed that 20.0% of millennials and 33.5% of individuals in generations Z had FGIDs. The most prevalent FGID among both generations was functional constipation, and type of beverages consumed was identified as the factor affecting the prevalence of FGIDs most in generation Z but not in millennials. FGIDs showed no association with COVID-19 infection in either generation. Interestingly, both generations were found to practice CAM to prevent diseases and enhance immunity. These findings can direct public health campaigns to raise awareness of the general population about FGIDs and the associated lifestyle factors that can prevent symptoms and promote a healthy lifestyle for both generations

## Figures and Tables

**Table 1:** Sample size summary.

	Total Population	Calculated Sample Size	Actual Sample Size
No. of eligible students	153,641 *	245 ^†^	376
No. of schools	566	12	8

* Total number of students in the academic year 2021–2022. ^†^ Minimal sample size required for the present study. Source: Statistics Department at the Ministry of Education [17]

**Table 2:** Prevalence of FGIDs among the sample.

Prevalence of FGIDs	Irritable Bowel Syndrome	Abdominal Migraine	Functional Abdominal Pain-Nos	Functional Constipation
Parents, *n* (%)	0	0	1 (0.7)	27 (19.2)
Students, *n* (%)	1 (0.2)	2 (0.5)	21 (5.5)	103 (27.3)

Abbreviations: FGIDs, functional gastrointestinal disorders; nos, not otherwise specified.

**Table 3:** Relationship between lifestyle factors affecting functional gastrointestinal diseases among the parents (*n* = 140).

Variable	Cases No. (%)	Controls No. (%)	*p* Value
Infected with COVID-19 previously			
Yes	7 (17.1)	34 (82.9)	0.57
No	21 (21.2)	78 (78.8)	
Exercises regularly			
Yes	16 (25.4)	47 (74.6)	0.14
No	12 (15.6)	65 (84.4)	
Practices CAM			
Yes	24 (20.9)	91 (79.1)	0.58
No	4 (16.0)	21 (84.0)	
Commonly consumed meals			
Home meals	27 (20.9)	102 (79.1)	0.34
Ready meals	1 (9.1)	10 (90.9)	
Dining out			
Yes	25 (23.4)	82 (76.6)	0.07
No	3 (9.2)	30 (90.9)	
Number of takeout meals per day			
None	3 (9.2)	30 (90.9)	0.30
1	17 (25.0)	51 (75.0)	
2	7 (21.2)	26 (78.8)	
3	1 (16.7)	5 (83.3)	

Data were analysed using chi-square test.

**Table 4:** Relationship between dietary factors affecting functional gastrointestinal diseases among the parents (*n* = 140).

Variable	Cases No. (%)	Controls No. (%)	*p* Value
Type of restaurants			
Does not eat in restaurants	3 (9.1)	30 (90.9)	0.17
Fast food	12 (25.5)	35 (74.5)	
Casual dining	13 (21.7)	47 (78.3)	
Type of commonly consumed beverages			
Water	18 (24.7)	55 (75.3)	0.36
Hot drinks	5 (12.8)	34 (87.2)	
Fresh fruit juice	3 (27.3)	8 (72.7)	
Sugar-sweetened carbonated drinks	2 (20.0)	80 (80.0)	
Sugar-free carbonated drinks	0 (0.0)	100 (100.0)	
Sugar-sweetened drinks	28 (20.0)	112 (80.0)	
Cups of water consumed per day			
Does not drink water	0 (0.0)	0 (0.0)	0.15
1	1 (50.0)	1 (50.0)	
2–3	7 (15.6)	38 (84.4)	
4–6	9 (15.5)	49 (84.5)	
7 or more	11 (31.4)	24 (68.6)	

Data were analysed using chi-square test. Abbreviations: CAM, complementary and alternative medicine.

**Table 5:** Relationship between lifestyle factors affecting functional gastrointestinal diseases among students (*n* = 376).

Variable	Cases No. (%)	Controls No. (%)	*p* Value
Infected with COVID-19 previously			
Yes	38 (38.0)	62 (62.0)	0.26
No	88 (31.9)	188 (68.1)	
Exercises regularly			
Yes	36 (27.1)	97 (72.9)	0.05
No	90 (37.0)	153 (63.0)	
Practices CAM			
Yes	87 (34.3)	167 (65.7)	0.58
No	38 (31.4)	83 (68.6)	
Commonly consumed meals			
Home meals	64 (28.3)	162 (71.7)	0.00 †
Ready meals	62 (41.3)	88 (58.7)	
Dining out			
Yes	112 (34.6)	212 (65.4)	0.27
No	14 (26.9)	38 (73.1)	
Number of takeout meals per day			
None	15 (28.3)	38 (71.7)	0.04 *
1	42 (27.5)	111 (72.5)	
2	49 (38.3)	79 (61.7)	
3	20 (47.6)	22 (52.4)	

Data were analysed using chi-square test. * *p* ≤ 0.05. † *p* ≤ 0.01

**Table 6:** Relationship between dietary factors affecting functional gastrointestinal diseases among students (*n* = 376).

Variable	Cases No. (%)	Controls No. (%)	*p* Value
Type of restaurants			
Does not eat in restaurants	14 (26.9)	38 (73.1)	0.41
Fast food	73 (33.2)	147 (66.8)	
Casual dining	39 (37.5)	65 (62.5)	
Type of commonly consumed beverages			
Water	50 (27.5)	132 (72.5)	0.03 *
Hot drinks	14 (34.1)	27 (65.9)	
Fresh fruit juice	10 (31.3)	22 (68.8)	
Sugar-sweetened carbonated drinks	29 (36.7)	50 (63.3)	
Sugar-free carbonated drinks	10 (52.6)	9 (47.4)	
Sugar-sweetened drinks	13 (56.5)	10 (43.5)	
Cups of water consumed per day			
Does not drink water	1 (100.0)	0 (0.0)	0.07
1	16 (48.5)	17 (51.5)	
2–3	47 (34.6)	89 (65.4)	
4–6	35 (26.5)	97 (73.5)	
7 or more	27 (36.5)	47 (63.5)	

Data were analysed using chi-square test. Abbreviations: CAM, complementary and alternative medicine. * *p* ≤ 0.05.

**Table 7:** Multinomial regression analysis for predictors of FGIDs among students (*n* = 376).

Variable	*p* Value	OR	95% CI
Exercise	0.12	1.45	0.90–2.35
Commonly consumed meals	0.09	1.48	0.93–2.35
Number of takeout meals per day	0.06	1.27	0.98–1.65
Types of commonly consumed beverages	0.01 *	1.18	1.03–1.35
Cups of water consumed per day	0.95	0.99	0.76–1.28

Data were analysed using multinomial regression analysis. Abbreviations: FGIDs, functional gastrointestinal diseases; OR: odds ratio; CI: confidence interval. * *p* ≤ 0.05.

## Data Availability

All new data were published along this article.

## References

[1] Mearin F., Malfertheiner P. (2018). Functional Gastrointestinal Disorders: Complex Treatments for Complex Pathophysiological Mechanisms. Dig. Dis..

[2] Black C.J., Drossman D.A., Talley N.J., Ruddy J., Ford A.C. (2020). Functional gastrointestinal disorders: Advances in understanding and management. Lancet.

[3] Drossman D.A., Thompson W.G., Talley N.J., Funch-Jensen P., Janssens J., Whitehead W.E. (1990). Identification of sub-groups of functional gastrointestinal disorders. Gastroenterol. Int..

[4] Kundur R., Lingala K.V.R., Alrshedi A.R.M. (2018). A study on the effect of dietary factors on functional gastrointestinal disorders in women of Ha’il region in Saudi Arabia. Asian J. Pharm. Clin. Res..

[5] Alshehri D.B., Sindi H.H., AlMusalami I.M., Rozi I.H., Shagrani M., Kamal N.M., Alahmadi N.S., Alfuraikh S.S., Vandenplas Y. (2022). Saudi Experts Consensus on Diagnosis and Management of Pediatric Functional Constipation. Pediatr. Gastroenterol. Hepatol. Nutr..

[6] Khayat A., Aldharman S.S., Alharbi N.N., Alayyaf A.S., Abdulmuttalib J.A., Altalhi E.R. (2023). Regional and seasonal variations in functional abdominal pain and functional constipation prevalence among Saudi children. SAGE Open Med..

[7] Khayat A., Algethami G., Baik S., Alhajori M., Banjar D. (2021). The Effect of Using Rome IV Criteria on the Prevalence of Functional Abdominal Pain Disorders and Functional Constipation among Children of the Western Region of Saudi Arabia. Glob. Pediatr. Health.

[8] Khatib M.A., Aljaaly E.A. (2023). Testing the Arabic-Saudi Arabia version of the Rome IV Diagnostic Questionnaire for functional gastrointestinal disorders for Children living in Saudi Arabia. Front. Pediatr..

[9] Wani F., Almaeen A., Bandy A., Thirunavukkarsu A., Al-Sayer T., Flah A., Fayed K., Albalawi M. (2020). Prevalence and risk factors of ibs among medical and nonmedical students in the jouf university. Niger. J. Clin. Pract..

[10] Wang M.-K., Yue H.-Y., Cai J., Zhai Y.-J., Peng J.-H., Hui J.-F., Hou D.-Y., Li W.-P., Yang J.-S. (2021). COVID-19 and the digestive system: A comprehensive review. World J. Clin. Cases.

[11] Farello G., Di Lucia A., Fioravanti B., Tambucci R., Stagi S., Gaudino R. (2021). Analysis of the impact of COVID-19 pandemic on functional gastrointestinal disorders among paediatric population. Eur. Rev. Med. Pharmacol. Sci..

[12] Cole G., Lucas L. (2002). The Debut of Generation y in the American Workforce. https://www.atu.edu/business/jbao/fall2002/cole_smith_lucas.pdf.

[13] Djafarova E., Foots S. (2022). Exploring ethical consumption of generation Z: Theory of planned behaviour. Young Consum..

[14] Banjar O., Ford-Gilboe M., Wong C., Befus D., Alilyyani B. (2022). The Association between Intimate Partner Violence and Functional Gastrointestinal Disorders and Symptoms among Adult Women: Systematic Review. J. Fam. Violence.

[15] (2022). Ministry of Education, Educational Offices in Jeddah Province [Internet]. https://sites.moe.gov.sa/Jeddah/workplace/.

[16] Al-Jaaly E.A. (2012). Factors Affecting Nutritional Status and Eating Behaviours of Adolescent Girls in Saudi Arabia. https://discovery.ucl.ac.uk/id/eprint/1370576/2/AL-Jaaly.1370576.Redacted__PhD_thesis.pdf.

[17] (2022). Ministry of Education, Statistics of Governmental Education. [Internet]. https://departments.moe.gov.sa/Statistics/Educationstatistics/Pages/GEStats.aspx.

[18] Bakhsh M.A., Khawandanah J., Naaman R.K., Alashmali S. (2021). The impact of COVID-19 quarantine on dietary habits and physical activity in Saudi Arabia: A cross-sectional study. BMC Public Health.

[19] Aljaaly E.A., Alhijri R., Al Nasser L. (2022). COVID-19 Effect on Dietary Supplements’ Consumption, Prophetic Medicine Practices and Herbs Use in Saudi Arabia. World Fam. Med. J. Middle East J. Fam. Med..

[20] Khatib M.A. (2022). The impact of Ramadan during COVID-19 confinement on weight, dietary, and lifestyle habits in the Kingdom of Saudi Arabia: A cross-sectional study. BMC Public Health.

[21] The Rome Foundation, Welcome to The Rome Foundation—Start Here. https://theromefoundation.org/#0.

[22] Lachat C., Hawwash D., Ocké M.C., Berg C., Forsum E., Hörnell A., Larsson C.L., Sonestedt E., Wirfält E., Åkesson A. (2016). Strengthening the Reporting of Observational Studies in Epidemiology—nutritional epidemiology (STROBE-nut): An extension of the STROBE statement. Nutr. Bull..

[23] El-Fetoh N.M.A., El-Mawgod M.M.A., Mohammed N.A., Alruwaili H.S.A., Alanazi E.O.M. (2016). Irritable Bowel Syndrome among Medical and Non-Medical Northern Border University Students, Kingdom of Saudi Arabia: Across Sectional Study. Open J. Gastroenterol..

[24] Ibrahim N.K.R., Battarjee W.F., Almehmadi S.A. (2013). Prevalence and predictors of irritable bowel syndrome among medical students and interns in King Abdulaziz University, Jeddah. Libyan J. Med..

[25] Murphy S.A., Weippert M.V., Dickinson K.M., Scourboutakos M.J., L’Abbé M.R. (2020). Cross-Sectional Analysis of Calories and Nutrients of Concern in Canadian Chain Restaurant Menu Items in 2016. Am. J. Prev. Med..

[26] Du Y., Rong S., Sun Y., Liu B., Wu Y., Snetselaar L.G., Wallace R.B., Bao W. (2021). Association between Frequency of Eating Away-from-Home Meals and Risk of All-Cause and Cause-Specific Mortality. J. Acad. Nutr. Diet..

[27] Malik V.S., Li Y., Pan A., De Koning L., Schernhammer E., Willett W.C., Hu F.B. (2019). Long-Term Consumption of Sugar-Sweetened and Artificially Sweetened Beverages and Risk of Mortality in US Adults. Circulation.

[28] Ebrahimi-Mameghani M., Sabour S., Khoshbaten M., Arefhosseini S.R., Saghafi-Asl M. (2017). Total diet, individual meals, and their association with gastroesophageal reflux disease. Health Promot. Perspect..

[29] Singh S.N., Singh A. (2015). Dietary fiber content of indian diets. Asian J. Pharm. Clin. Res..

[30] Surdea-Blaga T., Negrutiu D.E., Palage M., Dumitrascu D.L. (2019). Food and Gastroesophageal Reflux Disease. Curr. Med. Chem..

[31] Khodarahmi M., Azadbakht L., Daghaghzadeh H., Feinle-Bisset C., Keshteli A.H., Afshar H., Feizi A., Esmaillzadeh A., Adibi P. (2016). Evaluation of the relationship between major dietary patterns and uninvestigated reflux among Iranian adults. Nutrition.

[32] Kaltenbach T., Crockett S., Gerson L.B. (2006). Are lifestyle measures effective in patients with gastroesophageal reflux disease? An evidence-based approach. Arch. Intern. Med..

[33] Rao S.S.C., Kavelock R., Beaty J., Ackerson K., Stumbo P. (2000). Effects of fat and carbohydrate meals on colonic motor response. Gut.

[34] von Schönfeld J., Evans D.F., Renzing K., Castillo F.D., Wingate D.L. (1998). Human small bowel motor activity in response to liquid meals of different caloric value and different chemical composition. Dig. Dis. Sci..

[35] Khodarahmi M., Azadbakht L., Daghaghzadeh H., Feinle-Bisset C., Keshteli A.H., Afshar H., Feizi A., Esmaillzadeh A., Adibi P. (2004). Obesity is associated with increased risk of gastrointestinal symptoms: A population-based study. Am. J. Gastroenterol..

[36] Gupta S., Hawk T., Aggarwal A., Drewnowski A. (2019). Characterizing ultra-processed foods by energy density, nutrient density, and cost. Front. Nutr..

[37] Alasqah I., Mahmud I., East L., Usher K. (2021). Patterns of physical activity and dietary habits among adolescents in Saudi Arabia: A systematic review. Int. J. Health Sci..

[38] Almadi M., Almousa M., Althwainy A.F., Altamimi A.M., Alamoudi H., Alshamrani H.S., Alharbi O.R., Azzam N., Sadaf N., Aljebreen A.M. (2014). Prevalence of symptoms of gastroesopahgeal reflux in a cohort of Saudi Arabians: A study of 1265 subjects. Saudi J. Gastroenterol..

[39] Yönem Ö., SIiłvrił B., ÖzdemiIłr L., NadiIłr I., Yüksel S., Uygun Y. (2013). Gastroesophageal reflux disease prevalence in the city of Sivas. Turk. J. Gastroenterol..

[40] Fikree A., Byrne P. (2021). Management of functional gastrointestinal disorders. Clin. Med. J. R. Coll. Physicians Lond..

[41] Wilson K., Hill R.J. (2014). The role of food intolerance in functional gastrointestinal disorders in children. Aust. Fam. Physician.

[42] Pasqui F., Poli C., Colecchia A., Marasco G., Festi D. (2015). Adverse food reaction and functional gastrointestinal disorders: Role of the dietetic approach. J. Gastrointest. Liver Dis..

[43] Chey W.D. (2013). The Role of Food in the Functional Gastrointestinal Disorders: Introduction to a Manuscript Series. Am. J. Gastroenterol..

[44] Hasosah M., Alsahafi A., Alghiribi A., Alqarni N., Babatin A., Matrafi A., Alamri A., Qurashi M., Atiah N., Sarkhy A.A. (2018). Prevalence, characterization and risk factors of chronic constipation among saudi children: A cross-sectional study. Int. J. Adv. Res..

[45] Alhassan M., Alhassan A., Alfarhood A., Alotaibi K., Alrashidy N., Alshalhoub K., Almeshal M. (2019). Prevalence of constipation among central region population, Riyadh and Qassim provinces, Saudi Arabia, 2018–2019. J. Fam. Med. Prim. Care.

[46] Ali M., Almuqati B., Alhasnani H., Alfahmi T., Mandili A., Shatla M. (2021). The prevalence and risk factors of constipation among the general population in Makkah, Saudi Arabia. Int. J. Med. Dev. Ctries..

[47] Kruis W., Forstmaier G., Scheurlen C., Stellaard F., Kruis W. (1991). Effect of diets low and high in refined sugars on gut transit, bile acid metabolism, and bacterial fermentation. Gut.

[48] Rollet M., Bohn T., Vahid F. (2022). Association between Dietary Factors and Constipation in Adults Living in Luxembourg and Taking Part in the ORISCAV-LUX 2 Survey. Nutrients.

[49] Rodriguez D.A., Popov J., Ratcliffe E.M., Monjaraz E.M.T. (2021). Functional Constipation and the Gut Microbiome in Children: Preclinical and Clinical Evidence. Front. Pediatr..

[50] Zhu L., Liu W., Alkhouri R., Baker R.D., Bard J.E., Quigley E.M., Baker S.S. (2014). Structural changes in the gut microbiome of constipated patients. Physiol. Genom..

[51] de Moraes J.G., Motta M.E.F.D.A., Beltrão M.F.D.S., Salviano T.L., da Silva G.A.P. (2016). Fecal Microbiota and Diet of Children with Chronic Constipation. Int. J. Pediatr..

[52] de Meij T.G.J., de Groot E.F.J., Eck A., Budding A.E., Kneepkens C.M.F., Benninga M.A., van Bodegraven A.A., Savelkoul P.H.M. (2016). Characterization of microbiota in children with chronic functional constipation. PLoS ONE.

[53] Bhattarai Y., Williams B.B., Battaglioli E.J., Whitaker W.R., Till L., Grover M., Linden D.R., Akiba Y., Kandimalla K.K., Zachos N.C. (2018). Gut Microbiota-Produced Tryptamine Activates an Epithelial G-Protein-Coupled Receptor to Increase Colonic Secretion. Cell Host Microbe.

[54] Dass N.B., John A.K., Bassil A.K., Crumbley C.W., Shehee W.R., Maurio F.P., Moore G.B.T., Taylor C.M., Sanger G.J. (2007). The relationship between the effects of short-chain fatty acids on intestinal motility in vitro and GPR43 receptor activation. Neurogastroenterol. Motil..

[55] Vincent A.D., Wang X.-Y., Parsons S.P., Khan W.I., Huizinga J.D. (2018). Abnormal absorptive colonic motor activity in germ-free mice is rectified by butyrate, an effect possibly mediated by mucosal serotonin. Am. J. Physiol. Liver Physiol..

[56] Zhang D., Chen C., Xie Y., Zeng F., Chen S., Chen R., Zhang X., Huang S., Li D., Bai F. (2023). Post-infection functional gastrointestinal disorders following coronavirus disease-19: A prospective follow-up cohort study. BMC Infect. Dis..

[57] Nazarewska A., Lewandowski K., Kaniewska M., Tulewicz-Marti E., Więcek M., Szwarc P., Rosołowski M., Marlicz W., Rydzewska G. (2023). Long-lasting dyspeptic symptoms—Another consequence of the COVID-19 pandemic?. Gastroenterol. Rev..

[58] Al Akeel M., Al Ghamdi W., Al Habib S., Koshm M., Al Otaibi F. (2018). Herbal medicines: Saudi population knowledge, attitude, and practice at a glance. J. Fam. Med. Prim. Care.

[59] Holtmann G., Talley N.J. (2015). Herbal Medicines for the Treatment of Functional and Inflammatory Bowel Disorders. Clin. Gastroenterol. Hepatol..

[60] Madisch A., Holtmann G., Mayr G., Vinson B., Hotz J. (2004). Treatment of Functional Dyspepsia with a Herbal Preparation. Digestion.

[61] Rich G., Shah A., Koloski N., Funk P., Stracke B., Köhler S., Holtmann G. (2017). A randomized placebo-controlled trial on the effects of Menthacarin, a proprietary peppermint- and caraway-oil-preparation, on symptoms and quality of life in patients with functional dyspepsia. Neurogastroenterol. Motil..

[62] von Arnim U., Peitz U., Vinson B., Gundermann K.-J., Malfertheiner P. (2007). STW 5, a Phytopharmacon for Patients with Functional Dyspepsia: Results of a Multicenter, Placebo-Controlled Double-Blind Study. Am. J. Gastroenterol..

[63] Holtmann G., Schrenk D., Madisch A., Allescher H.D., Ulrich-Merzenich G., Mearin F., Larrey D., Malfertheiner P. (2020). Use of Evidence-Based Herbal Medicines for Patients with Functional Gastrointestinal Disorders: A Conceptional Framework for Risk-Benefit Assessment and Regulatory Approaches. Dig. Dis..

[64] Kim Y.S., Kim J.W., Ha N.Y., Kim J., Ryu H.S. (2020). Herbal Therapies in Functional Gastrointestinal Disorders: A Narrative Review and Clinical Implication. Front. Psychiatry.

